# Mechanical oscillation accelerating nucleation and nuclei growth in hard-sphere colloidal glass

**DOI:** 10.1038/s41598-019-49120-1

**Published:** 2019-09-06

**Authors:** Nobutomo Nakamura, Shizuka Nakashima, Hirotsugu Ogi

**Affiliations:** 1 Graduate School of Engineering Science, Osaka University, 1-3 Machikaneyama, Toyonaka, Osaka 560-8531 Japan; 2 Graduate School of Engineering, Osaka University, 2-1 Yamadaoka, Suita, Osaka 565-0871 Japan

**Keywords:** Structure of solids and liquids, Colloids

## Abstract

Crystallization from amorphous solids is generally caused by activating phonons in a wide frequency range during heat treatment. In contrast, the activation of phonons in a narrow frequency range using ultrasonic treatment also causes crystallization below the glass transition temperature. These behaviors indicate that crystallization is related to the atomic motion in the glass state, and it is suggested that the activation of specific atomic motion can cause crystallization without increasing temperature. In this study, we observe nucleation and nuclei growth caused by mechanical oscillation in a hard-sphere colloidal glass and evaluate the effect of mechanical oscillation on the structural evolution in the early stage of the crystallization. Oscillation between 5 and 100 Hz is applied to the colloidal glass, and it is observed that the nucleation rate increases under the 70 Hz oscillation, resulting in formation of stable nuclei in a short amount of time. The nuclei growth is also accelerated by the 70 Hz oscillation, whereas increases in the nucleation rate and nuclei growth were not observed at other frequencies. Finally, activation of the diffusion-based rattling of particles by caging is considered as a possible mechanism of the observations.

## Introduction

Carefully controlled heat treatment is a common technique used to crystallize amorphous solids and produce nanocrystalline materials that possess characteristic mechanical and magnetic properties^1–4^. During heat treatment, phonons in a wide-frequency range are activated. In comparison, the excitation of mechanical vibrations in a narrow frequency range can also cause crystallization. In Pd-based metallic glass, ultrasonic irradiation leads to crystallization below the glass transition temperature^5^. This phenomenon is explained by specific atomic motion that is stochastically resonant with the ultrasonic vibrations; the activation leads to crystallization. This result indicates that specific atomic motion contributes to crystallization in amorphous solids, and activation of the motion causes crystallization without increasing the temperature. Achieving crystallization by using ultrasonic irradiation would be advantageous over heat treatment because it can selectively crystallize desired regions. However, oscillation-induced crystallization from amorphous solids has barely been investigated.

In glass forming materials, characteristic atomic motion exists, e.g., *α* relaxation, *β* relaxation, and boson peak^6,7^. Activating such atomic motion is considered to be a possible cause of accelerated crystallization. In Pd-based metallic glass, the activation of atomic motion related to *β* relaxation caused crystallization^5^. The time scale of the characteristic atomic motions varies widely, and it becomes as small as 10^−15^ s for motion related to boson peak. Therefore, it is difficult to activate atomic motion selectively and to evaluate the relationship between the oscillation frequency and crystallization in experiments.

In this study, we focus on a hard-sphere colloidal glass. Colloids composed of nearly hard colloidal spheres and a solution exhibit phases that are observed in atomic materials^8^, and the glass phase, the colloidal glass, has been used for simulating atomic-scale motion in glass materials^9–14^. In colloidal systems, particle coordinates can be measured by using a confocal microscope, and the time scale of the particle motion is significantly longer than that in atomic systems. In addition, colloidal systems comprising more than a billion particles can be used. These features are suitable for analyzing atomic-scale dynamics in amorphous solids.

The effect of applying mechanical oscillations on colloidal systems has been studied^15–17^, and the relationship between the oscillation frequency and crystallization was investigated^18,19^. When oscillatory shears were applied in colloidal gels at 1, 10, and 70 Hz, the strain amplitude required for yielding/crystallization decreased with increasing oscillation frequency^18^. In that study, a model is proposed for explaining the results, in which crystallization occurs when a time scale that is calculated from the frequency and amplitude becomes smaller than a specific value. The model indicates that the crystallization occurs when the applied frequency becomes larger than a specific frequency with constant amplitude. In contrast, in a colloidal glass, crystallization was accelerated by oscillations around 70 Hz; below and above the frequency, crystallization hardly occurred^19^. The crystallization frequency increased by increasing the inter-particle attractive force, and it implies that there are specific particle motions contributing to crystallization. This phenomenon cannot be explained by the above described model, indicating different phenomenon was found. However, its mechanism was not clarified.

In crystallization process, nuclei are formed initially, and after stable nuclei are formed nuclei growth occurs. Therefore, crystallization is accelerated by activating the nucleation and/or nuclei growth. In the previous study^19^, structural evolution after applying 5-s oscillations was observed. In the measurement, the crystallization progressed quickly, and a large part of the colloidal glass was crystalized after the 5-s oscillation. Therefore, nucleation and nuclei growth were not observed; the duration time, 5 s, was too long to observe the structural evolution in the early stage of nucleation. In this study, we focus on the early stage of crystallization, and investigate whether the mechanical oscillation contributes to nucleation or nuclei growth by reducing the duration time of the oscillation. Then, a possible mechanism of the oscillation induced crystallization is discussed.

## Results and Discussion

The measurement system that was developed in our previous study^19^ was used. We used a colloidal suspension consisting of a mixture of water and dimethyl sulfoxide (DMSO) with fluorescein sodium salt (FSS) and silica particles of 1.5 *μ*m diameter with polydispersity index less than 0.2. In the suspension, the refractive indices of the solvent and the particles were similar. A colloidal glass was formed by settling silica particles dispersed in solution on a bottom surface of a sample cell by centrifugation. In the following experiments, a new sample was prepared for each measurement. Mechanical oscillation was applied to the colloidal glass by moving an oscillator inserted into the colloidal suspension. The oscillator was not touching the colloidal glass. Structure of the colloidal glass was observed by using the confocal laser scanning microscope. Size of the microscope image was 71.7 × 71.7 *μ*m. Detail of the sample and measurement setup are described in Methods. In the following experiments, we analyze two-dimensional (2D) images. Three-dimensional (3D) images are helpful for analyzing the detailed structure of colloidal glass. However, it takes longer time to obtain 3D images, and fading and temperature increment that causes the structural change in the colloidal glass will occur. To remove these effects, we performed 2D analysis.

**Figure 1:**
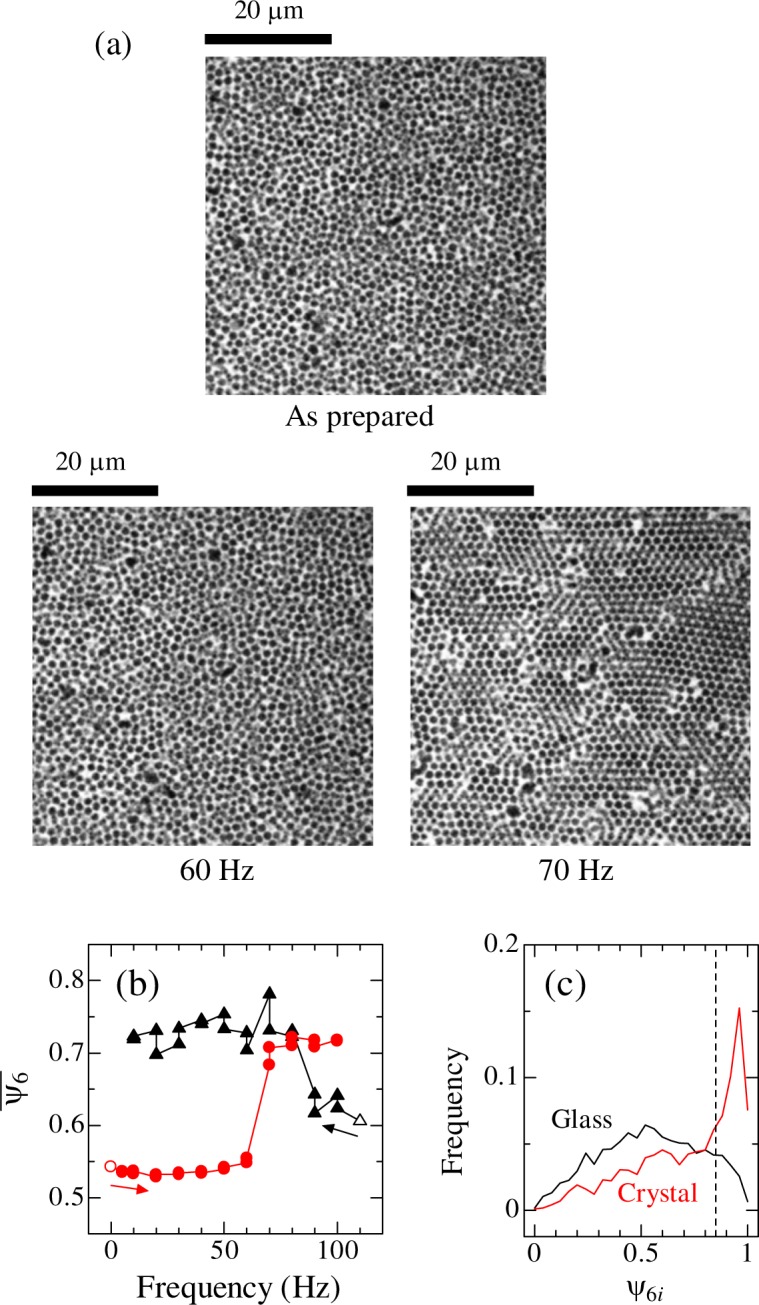
(**a**) Representative images taken before and after applying horizontal oscillations at 60 and 70 Hz. (**b**) Evolution of the averaged value of *ψ*_6*i*_. Oscillation frequency was varied from 5 to 100 Hz (circles) and from 100 to 10 Hz (triangles). Open symbols denote results of as-prepared samples. (**c**) Histograms of *ψ*_6*i*_ for an as-prepared sample (glass state) and for the corresponding sample after oscillation was applied from 5 to 100 Hz (crystalline state). The dashed line indicates *ψ*_6*i*_ = 0.85.

We applied horizontal (shear) oscillation to the colloidal glass and confirmed its effect on crystallization. The oscillation frequency was varied between 5 and 100 Hz. The oscillation amplitude of the oscillator was fixed at 5 *μ*m by using the closed-loop feedback of the piezo-stage. Oscillation with duration time of 5 s was applied twice, totally 10 s, at each frequency. 2D images were taken after applying 5 s of oscillation (two images per frequency). Figure 1(a) shows representative microscopy images taken during an experiment when the frequency was increased from 5 Hz to 100 Hz. When the oscillation frequency was 60 Hz or lower, notable crystallization was not observed. However, after 70 Hz oscillation was applied, crystallization occurred clearly.

To quantify the degree of crystallization, the 2D local bond orientational order parameter *ψ*_6*i*_^19,20^ was calculated for each particle. When the neighboring particles of a particle *i* show six-fold symmetry, *ψ*_6*i*_ of the particle becomes unity, and it becomes smaller as the structure becomes random. The averaged value ${\bar{\psi }}_{6}$ of *ψ*_6*i*_ over particles in each 2D image is plotted in Fig. 1(b). When the oscillation frequency was varied from 5 to 100 Hz, ${\bar{\psi }}_{6}$ remained nearly unchanged at 5–60 Hz. After 70 Hz oscillation was applied, ${\bar{\psi }}_{6}$ increased, and crystallization was clearly identified. When oscillation was applied to a colloidal glass with decreasing frequency from 100 to 10 Hz, the ${\bar{\psi }}_{6}$ increased after 80 Hz oscillation was applied (Fig. 1(b)). The frequencies at which the crystallization occurred were similar to that observed in our previous study^19^. From these results, it was confirmed that there is particle motion contributing to crystallization at approximately 70 Hz.

After 70 Hz oscillation was applied, a large part of the colloidal glass was crystallized. The oscillation time was too long to observe nucleation. Therefore, we reduced the oscillation time to 0.3 s and observed the structural evolution. Three samples were prepared for this experiment. Oscillation was applied to the samples at 30, 70, and 100 Hz, and 2D images were taken repeatedly after applying the 0.3 s long oscillation. The obtained images are shown in the Supplementary Videos 1–3. Image quality is lowered to reduce the file size.

**Figure 2:**
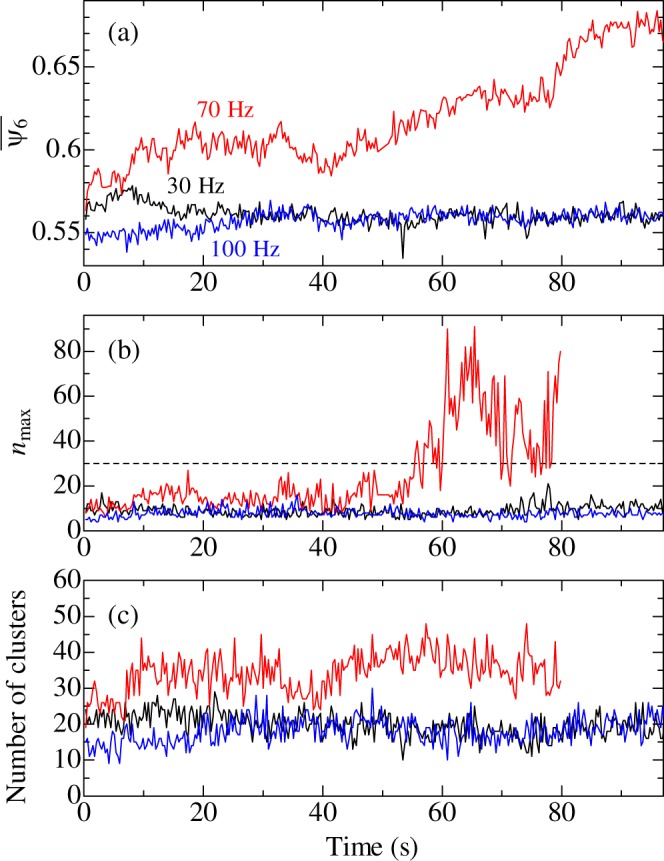
Evolutions of (**a**) averaged value of *ψ*_6*i*_, (**b**) the number of particles composing the largest crystalline cluster, and (**c**) the number of the crystalline clusters. A dashed line in (**b**) indicates *n*_max_ = 30.

The relationship between ${\bar{\psi }}_{6}$ and the oscillation time is plotted in Fig. 2(a). For 30 and 100 Hz oscillation, ${\bar{\psi }}_{6}$ did not vary with increasing oscillation time. However, when 70 Hz oscillation was applied, ${\bar{\psi }}_{6}$ increased gradually and was almost stable between 10 and ~40 s. After this period, ${\bar{\psi }}_{6}$ started to increase again, indicating that crystallization progressed.

The evolution of the crystalline cluster size was also evaluated. As seen in Fig. 1(c), after crystallization occurred, the proportion of particles whose *ψ*_6*i*_ is larger than 0.85 increased. These particles were considered to be crystallized particles and were extracted from the analyzed particles. Then, to remove the isolated particles and enhance the crystallized regions, particles having two or fewer neighboring particles were eliminated. The remaining particles (included in clusters) were plotted in a 2D image, and the number *n*_max_ of particles comprising the largest cluster in the image was counted.

The evolution of *n*_max_ is plotted in Fig. 2(b). For 30 and 100 Hz oscillation, *n*_max_ remained at approximately 10. When 70 Hz oscillation was applied, *n*_max_ increased slightly, and it started to increase markedly at ~50 s. In the theory of homogeneous crystallization, stable nuclei are formed when the diameter of the crystalline cluster becomes larger than the critical diameter^21^. In Fig. 2(b), *n*_max_ was smaller than 30 before nucleation occurred, thus indicating that the critical size of nuclei was larger than 30 particles, which corresponded to about 6 particles in diameter. At approximately 80 s, crystalline clusters came in contact with each other, and *n*_max_ was not counted.

In the 2D images, drift and changes in focal plane were observed. Their cause is unknown. Regarding 30 and 100 Hz oscillation, the 2D images around the initial focal area were obtained due to the drift and the changes in the focal plane. In the images, notable crystallization was not observed, and it confirmed that crystalline regions are not formed around the focal area. Regarding 70 Hz oscillation, some particles move in and out of the field, because particles move for creating crystalline regions. The motion is inevitable, and it may affect analysis of the structural evolution. However, the change in ${\bar{\psi }}_{6}$ in Fig. 2(a) can be visually confirmed in the 2D images (Supplementary Video 2). For these reasons, the drift and the change in the focal plane barely affect the above analysis.

**Figure 3:**
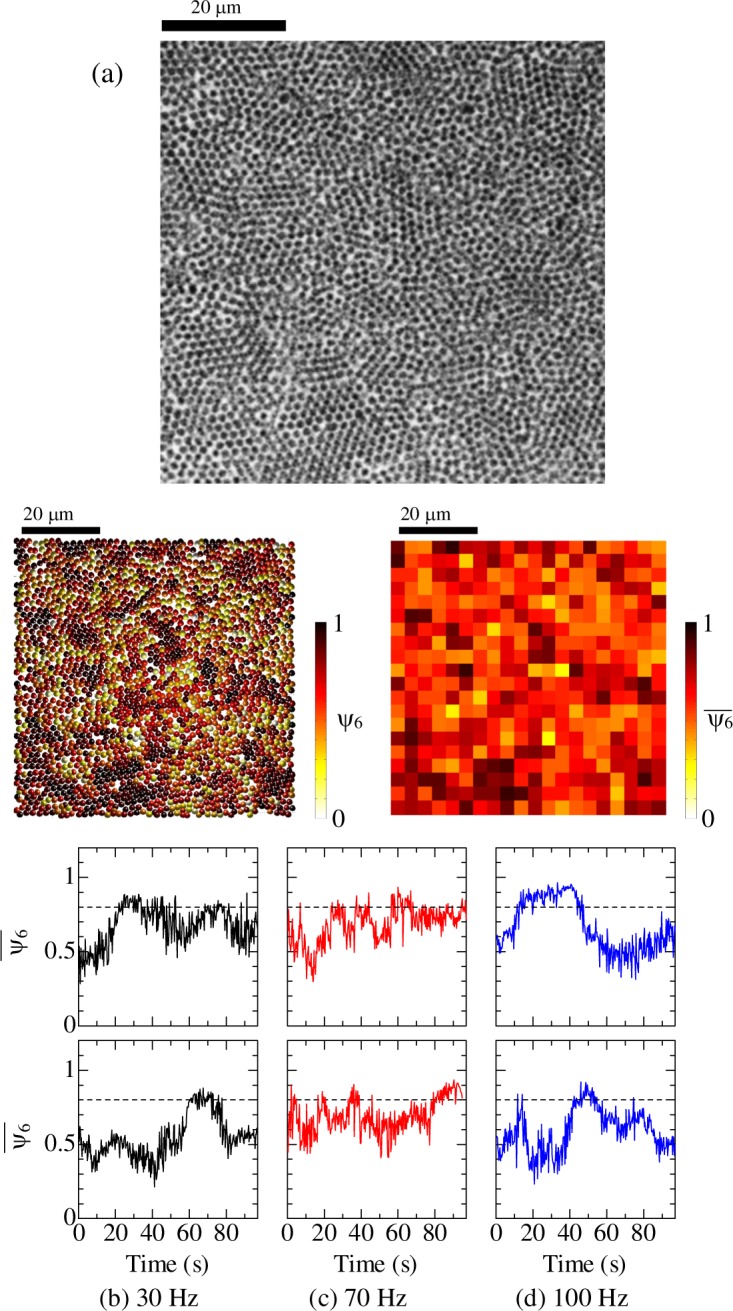
Evolution of ${\bar{\psi }}_{6}$ during the applications of mechanical oscillation to colloidal glasses. (**a**) A microscope image (top), a reconstructed image showing the distribution of *ψ*_6*i*_ (bottom left), and a reconstructed image showing ${\bar{\psi }}_{6}$ per square (bottom right). The evolutions of ${\bar{\psi }}_{6}$ of the representative two squares at (**b**) 30 Hz, (**c**) 70 Hz, and (**d**) 100 Hz are plotted. Dashed lines denote the threshold above which crystallization happened.

After above analysis, the nucleation rate was evaluated. 2D images were divided into 20 by 20 squares; the size of the square is 3.6 × 3.6 *μ*m. The average value of *ψ*_6*i*_ in each square was calculated as shown in Fig. 3(a). The value of ${\bar{\psi }}_{6}$ in squares where crystallization occurs can be smaller than the threshold value of 0.85 determined in Fig. 1(c), because particles with small *ψ*_6*i*_ are included in the squares. Therefore, 0.8 is considered as the threshold in this analysis. Figure 3(b–d) show evolution of ${\bar{\psi }}_{6}$ in representative two squares when 30, 70, and 100 Hz oscillation was applied, respectively. When 30 and 100 Hz oscillation was applied, nucleation occurred once or twice during the experimental period, but stable nuclei were not formed. This is attributed that the size of the nuclei (crystalline clusters) was smaller than the critical size. In contrast, when oscillation at 70 Hz was applied, nucleation occurred more frequently. As described above, crystallization markedly occurred around 50 s under the 70 Hz oscillation, but formation of crystalline clusters occurred frequently before the crystallization started. The drift in the 2D images may affect the above analysis. When crystalline regions move from an original square to a neighboring square, ${\bar{\psi }}_{6}$ of the original square decreases, whereas the crystalline regions exist in the 2D image. Therefore, actual duration time that nuclei exist may be longer than that observed in Fig. 3(b–d). However, it was obviously observed in the Supplementary Videos 1–3 that particle motion is strongly excited only by 70 Hz oscillation and structural change is accelerated. In Fig. 2(c), changes in the number of the crystalline clusters when the oscillations are applied are plotted. The number of the crystalline clusters under 70-Hz oscillation is larger than that under 30- and 100-Hz oscillation. This result also supports the view that the crystalline clusters are formed frequently at 70 Hz. Based on these results, we consider that oscillation at 70 Hz increased the rate of nucleation (formation of crystalline cluster) and accelerated the onset of nucleation.

**Figure 4:**
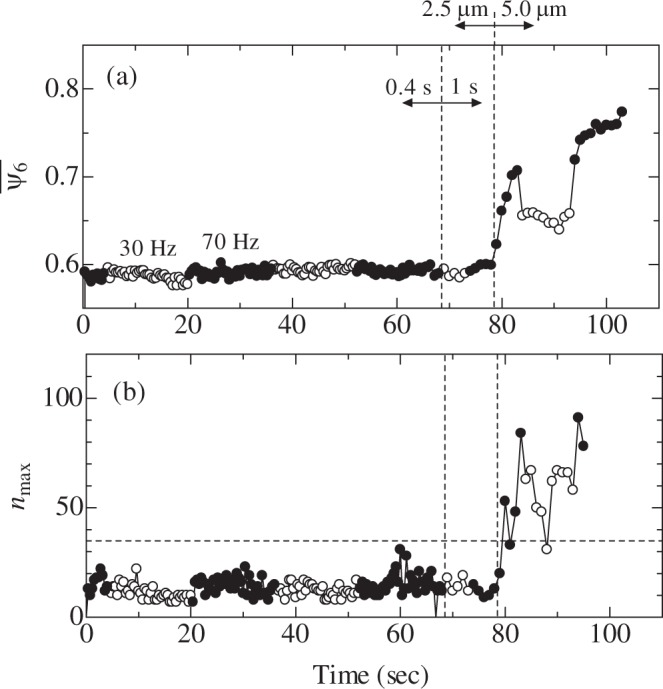
Evolutions of (**a**) ${\bar{\psi }}_{6}$ and (**b**) *n*_max_ when 30 and 70 Hz oscillations were applied alternately. Vertical dashed lines denote where oscillation time and amplitude were changed. A horizontal dashed line in (**b**) indicates *n*_max_ = 35.

To access whether the 70 Hz oscillation contributed to the growth of the nuclei as well as nucleation, oscillation was applied at 30 and 70 Hz alternately to a colloidal glass. The amplitude was 2.5 *μ*m and the oscillation time was 0.4 s. The results are shown in Fig. 4. During this sequence, ${\bar{\psi }}_{6}$ increased slightly under the 70 Hz oscillation, but notable crystallization was not observed (Fig. 4(a)). The amplitude and duration time were then changed to 5 *μ*m and 1 s, respectively, after which the crystallization progressed rapidly; a partially-crystallized colloidal glass was formed. When 30 Hz oscillation was applied to this sample, an increase in ${\bar{\psi }}_{6}$ was not observed, although crystal growth started again after subsequent application of 70 Hz oscillation. This result indicates that ~70 Hz oscillation contributes to both the nucleation and crystal growth, and oscillation at other frequencies does not contribute to them. The value of *n*_max_ was also determined for this case, as shown in Fig. 4(b). Before the amplitude was changed from 2.5 to 5.0 *μ*m, a notable increase was not observed, and *n*_max_ was smaller than 35. This value was close to that observed in Fig. 2(b), and it confirms that the diameter of minimum stable nuclei is about 6 particles. After the amplitude was increased, *n*_max_ increased over time under 70 Hz oscillation. However, *n*_max_ was stable under 30 Hz oscillation, which confirmed that the 70 Hz oscillation contributed to nuclei growth as well as nucleation. The images used in this analysis are shown in Supplementary Video 4. Image quality is lowered to reduce the file size.

In colloidal glass, individual particles are trapped in cages formed by their neighbors, and rattling of particles in the cages occurs. We here consider time scale of the rattling. In the present colloidal suspension, the solvent is a mixture of DMSO and water, and its viscosity *η* is deduced to be 3.64 mPa·s from the reported viscosity of a DMSO-water system^22^. When the volume fraction of the colloidal glass composed of 1.5-*μ*m particles is 0.59, which was measured in our previous work^19^, distance between the surface of neighboring particles is 0.041 *μ*m. This value is calculated assuming that particles contact completely at volume fraction of 0.64. From these parameters, the diffusion coefficient *D* (=*k*_*B*_*T*/6*πηa*) is calculated to be 8.0 × 10^−14^ m^2^/s at *T* = 300 K, and time Δ*t* (=〈Δ*x*^2^〉/2D) that the particle diffuses for 0.041 *μ*m to contact a neighboring particle becomes 0.01 s. Here, *a* is the radius of the colloidal particle. This time scale is close to the inverse of the frequency at which crystallization is accelerated, ~0.014 = 1/70 s. Therefore, activation of the diffusion-based rattling by caging of neighboring particles is a possible mechanism of our observation. The time scale of the rattling changes depending on the volume fraction of colloidal glass. For example, it becomes 0.007 s, when the volume fraction is 0.60 (distance between the surface of neighboring particles is 0.033 *μ*m). In our previous work^19^, we evaluated effect of the inter-particle attractive force on the crystallization frequency. Attractive force was induced through the depletion force by adding poly(sodium 4-styrensulfonate) (PSS) into the colloidal suspension. Then, crystallization frequency increased as the PSS concentration increased; the crystallization frequency was 70, 90, and 95–100 Hz at 0, 1.0, and 1.5 *μ*M, respectively. When the attractive force increases, the inter-particle distance decreases and the volume fraction increases, which should decrease the time scale of the diffusion-based rattling. Thus, the diffusion-based rattling can explain our experimental results.

**Figure 5:**
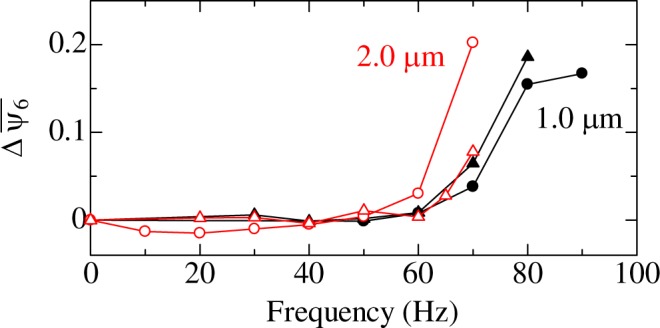
Changes in ${\bar{\psi }}_{6}$ for colloidal glasses composed of 1 *μ*m (filled symbols) and 2 *μ*m (open symbols) particles, when horizontal oscillation was applied to them for 60 s at each frequency.

According to the analysis above, time scale of the rattling depends on the particle size as well as the volume fraction, and it becomes smaller (frequency increases) as the particle becomes smaller. To confirm relationship between the diffusion-based rattling and crystallization phenomenon, we evaluated the dependence of the crystallization frequency on the particle size. Colloidal glasses composed of 1 *μ*m-diameter and 2 *μ*m-diameter particles were prepared, and horizontal oscillation was applied to them with increasing the frequency. Duration time of the oscillation was 60 s at each frequency. In this experiment, height of the colloidal glass was ~70 *μ*m. Relationship between the applied frequency and ${\bar{\psi }}_{6}$ is plotted in Fig. 5. The crystallization frequency for the colloidal glass composed of 1-*μ*m particle is 80 Hz, and it is larger than that for the colloidal glass composed of 2-*μ*m particle, 70 Hz. Although the difference is not large, the change in the frequency is expected by the diffusion-based rattling phenomenon. In the present colloidal system, mass density of silica particle is larger than that of the solution, and the force by the gravity affects the particle motion. In the system, a volume fraction gradient occurs, and its degree will change depending on the particle size. In addition, when the particle size is changed, the force by the gravity will affect the diffusive motion of particles. Therefore, the simple analytic model described above fails to explain the experimental result quantitatively. However, experimentally observed relationship between the crystallization frequency and particle size is qualitatively explained by the diffusion-based rattling phenomenon, indicating that the diffusion-based rattling is a possible cause of crystallization at a specific frequency.

## Conclusions

In conclusion, mechanical oscillation around 70 Hz induced crystallization in hard-sphere colloidal glass. The applied oscillation increased the nucleation rate, and nuclei growth was also activated, whereas noticeable increases in the nucleation rate and nuclei growth were not observed at other frequencies. In addition, the diameter of minimum stable nuclei in the colloidal glass was observed at about 6 particles. Finally, it was considered that the diffusion-based rattling in the cage formed by neighboring particles is related to the above results.

## Methods

**Figure 6:**
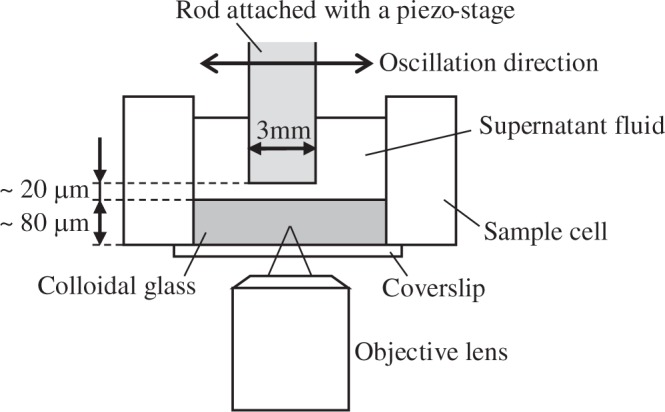
Schematic image of experimental setup.

We used colloidal suspensions consisting of a mixture of water (37.2% by volume) and dimethyl sulfoxide (62.8% by volume) with fluorescein sodium salt (FSS) and silica particles of 1.0, 1.5, and 2.0  *μ*m diameter with polydispersity index less than 0.2 (Micromod, sicastar). In the suspension, the refractive indices of the solvent and the particles were similar. FSS concentration was 2.0 mM, and the volume fraction of the colloidal particles was ~0.01. Addition of the FSS to the suspension shortens screening length, and nearly hard-sphere interaction is obtained^23^. A sample cell for microscopy observation was composed of a cover slip and an aluminum block with a through hole of 9-mm diameter. Particles with 5 *μ*m diameter were attached onto the coverslip using polymethyl methacrylate, and the coverslip was attached to the aluminum block (Fig. 6). The colloidal suspension was poured into a sample cell, and colloidal glass was formed on the coverslip by centrifuging the cell. Centrifugal acceleration was approximately 1850 *g*, where *g* = 9.81 m/s^2^. Creating a rough surface on a sample cell using large (5 *μ*m) particles is a technique used for preventing the crystallization on the coverslip, and it was used in previous shear experiments^12,24^. The colloidal particles were observed through the coverslip using a confocal laser scanning microscope (Nikon A1R). 60× oil immersion lens was used, and the wavelength of the excitation light was 488 nm.

Mechanical oscillation was applied to the colloidal glass using an aluminum rod with a square cross section of 3 × 3 mm^2^. After the sample cell was placed on the stage of the microscope, the rod was gently inserted into the suspension vertically using an XYZ stage. The height of the colloidal glass was approximately 80 *μ*m, and the gap between the top surface of the colloidal glass and the tip of the rod was approximately 20 *μ*m. The tip of the rod was not in contact with the colloidal glass. The rod was moved by a piezo-stage in the horizontal direction. Shear strain generated beneath the bottom surface of the rod deformed the colloidal glass, and structural variation beneath the rod was observed. Details of the sample cell and measurement setup are described elsewhere^19^. 2D images were taken with the microscope at 30 *μ*m above the coverslip surface. After smoothing the images by replacing each pixel with the neighborhood mean, the particles’ coordinates were determined using the previously reported algorithm^25^. About 2500 particles are included in the images, when 1.5 μm particle is used. The images provided in Supplementary Videos are the data we analyzed in the present study.

**Figure 7:**
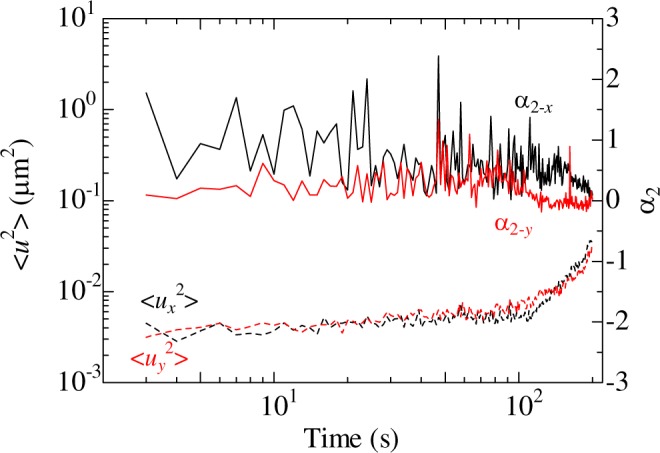
The one-dimensional mean square displacement (dashed lines) and nongaussian parameter (solid lines).

In previous studies, the phase of a colloidal suspension was evaluated by measuring the ensemble-averaged mean square displacement (MSD) and the nongaussian parameter *α*_2_^11,26^. Referring to ref.^11^, we calculated the one-dimensional MSD and *α*_2_ for a representative sample composed of 1.5 μm particles. The MSD and *α*_2_ are shown in Fig. 7. In the MSD, a plateau appears, followed by the gradual increase. The plateau indicates the cage trapping, and this behavior is similar to that observed in colloidal glasses in the previous study^11^. Regarding *α*_2_, it is reported to show a peak in fluids at a certain time, whereas it decreases with time in the colloidal glass. In Fig. 7, the peak was not observed, and *α*_2_ decreased gradually. Thus, it was confirmed that our sample shows relaxation behaviors that are observed in colloidal glasses.

## Supplementary information

Supporting video Fig.2 30 Hz

Supporting video Fig.2 70 Hz

Supporting video Fig.2 100 Hz

Supporting video Fig.4

## Data Availability

The datasets generated during and/or analysed during the current study are available from the corresponding author on reasonable request.
